# Molecular characterization of coxsackievirus B5 from the sputum of pneumonia children patients of Kunming, Southwest China

**DOI:** 10.1186/s12985-023-02019-w

**Published:** 2023-04-19

**Authors:** Miao Tan, Jiale Suo, Zhilei Zhang, Wenji He, Li Tan, Haiyan Jiang, Ming Li, Juan He, Yue Pan, Bin Xu, Lingmei Yan, Songtao Bin, Zhengyan Gan, Yuxing Sun, Hongchao Jiang, Qiangming Sun, Zhen Zhang

**Affiliations:** 1 Department of Clinical Pharmacy, Affiliated Hospital of Yunnan University, 176 Qing Nian Road, Kunming, 650118 Yunnan Province People’s Republic of China; 2 Institute of Medical Biology, Chinese Academy of Medical Sciences and Peking Union Medical College (CAMS & PUMC), 935 Jiao Ling Road, Kunming, 650118 Yunnan Province People’s Republic of China; 3 Kunming Children’s Hospital , The Affiliated Children’s Hospital of Kunming Medical University, 288 Qian Xin Road, Kunming, 650228 Yunnan Province People’s Republic of China; 4 Yunnan Key Laboratory of Children’s Major Disease Research, Kunming, People’s Republic of China; 5 Yunnan Key Laboratory of Vaccine Research and Development on Severe Infectious Diseases, Kunming, People’s Republic of China; 6 Kunming Medical University, Kunming, People’s Republic of China

**Keywords:** CVB5, Analysis, Pneumonia, Epidemiology

## Abstract

**Background:**

CVB5 can cause respiratory infections. However, the molecular epidemiological information about CVB5 in respiratory tract samples is still limited. Here, we report five cases in which CVB5 was detected in sputum sample of pneumonia children patients from Kunming, Southwest China.

**Methods:**

CVB5 isolates were obtained from sputum samples of patients with pneumonia. Whole-genome sequencing of CVB5 isolates was performed using segmented PCR, and phylogenetic, mutation and recombination analysis. The effect of mutations in the VP1 protein on hydration were analyzed by Protscale. The tertiary models of VP1 proteins were established by Colabfold, and the effect of mutations in VP1 protein on volume modifications and binding affinity were analyzed by Pymol software and PROVEAN.

**Results:**

A total of five CVB5 complete genome sequences were obtained. No obvious homologous recombination signals comparing with other coxsackie B viruses were observed in the five isolates. Phylogenetic analysis showed that the five CVB5 sputum isolates were from an independent branch in genogroup E. Due to the mutation, the structure and spatial of the VP1 protein N-terminus have changed significantly. Comparing to the Faulkner (CVB5 prototype strain), PROVEAN revealed three deleterious substitutions: Y75F, N166T (KM35), T140I (KM41). The last two of the three deleterious substitutions significantly increased the hydrophobicity of the residues.

**Conclusions:**

We unexpectedly found five cases of CVB5 infection instead of rhinoviruses infection during our routine surveillance of rhinoviruses in respiratory tract samples. All five patients were hospitalized with pneumonia symptoms and were not tested for enterovirus during their hospitalization. This report suggests that enterovirus surveillance in patients with respiratory symptoms should be strengthened.

## Introduction

*Enterovirus B* is considered to be a common respiratory pathogen in young children and can cause respiratory wheezing disease, including bronchiolitis and exacerbation of asthma [1, 10, 16, 17]. CVB5 belongs to the species *Enterovirus B* of the Picornaviridae family [5, 9, 23] and the genomic RNA is about 7.5 kb long in length and encodes a large polyprotein, which consists of the structural proteins (VP1-4) and other nonstructural proteins [15]. CVB5 uses DAF as a receptor for virus attachment to cells and it depends on CAR for virus entry and virus replication processes. Aseptic meningitis and viral encephalitis caused by CVB5 occur worldwide, with outbreaks or epidemics reported in Europe, North America, South America and Asia [6]. The first reported clinical symptom of CVB5 was lower respiratory tract infection, and in recent years, the clinical symptoms of CVB5 have included HFMD [24]. CVB5 usually occurs in summer and autumn and the susceptible populations are mainly under 18 years old. To date, the vaccines against enteroviruses are those for poliovirus and EV-A71 [23]. The two vaccines offer no cross-protection against other enteroviruses, and there are no vaccines or specific drugs against CVB5 [4]. Therefore, the study of CVB5 has become an urgent need for respiratory pathogen research and respiratory disease prevention.

We unexpectedly found five cases of CVB5 infection during routine surveillance of rhinoviruses in respiratory tract samples. All five patients were hospitalized with pneumonia symptoms and were not tested for enterovirus and rhinovirus during their hospitalization. But we found CVB5 infection instead of rhinovirus infection in their sputum samples through follow-up experiments. Considering the molecular epidemiological information on respiratory tract samples containing CVB5 is still limited and clinicians lack attention to the detection of enterovirus in patients with respiratory symptoms, we decided to study it. In this study, we obtained five complete genome sequences from the sputum of inpatients in the respiratory department of the Children’s Hospital Affiliated with Kunming Medical University. Meanwhile, nine CVB5 VP1 sequences have been obtained by our team from the feces of patients with HFMD during a large enterovirus infection outbreak in Kunming in 2018 [11, 18]. To explore the molecular characteristics of CVB5 in patients with different symptoms, transmission and evolution of CVB5 in Kunming, we compared these sequences and performed phylogenetic, mutation and recombination analysis, structural analysis and prediction. Our study may benefit the research of the genetic characterization, potential source and evolution of CVB5.

## Materials and methods

### Sample collection

A total of 108 sputum samples of patients with respiratory tract inpatients were collected from the Children’s Hospital Affiliated with Kunming Medical University. This hospital is an academic, tertiary care paediatric hospital with > 1200 inpatient beds, and the hospital treats more than 2 million patients every year.

### RNA extraction and CVB5 detection

Virus isolation was performed by inoculation into the human cervical cancer cells (ATCC CRL-1958). The Omega Viral RNA Kit (Omega Biotek, United States) was used for viral RNA extraction, and viral RNA extraction was performed in accordance with the manufacturer’s instructions. The extracted viral RNA was stored at − 80 °C. Using the primer of the 5′-UTR of rhinovirus amplified the 394 bp fragment genome by RT-PCR. A Prime Script™ RT reagent Kit with gDNA Eraser (Code No. RR047A, Takara BioInc, Beijing, China) and Premix Taq™ Ex Taq™ Version 2.0 plusdye (Code No. RR902A, Takara Bio Inc, Beijing, China) was used for RT-PCR (http://www.takarabiomed.com.cn). The PCR products were confirmed by agarose gel electrophoresis and sent to Tsingke Biotech (Beijing, China) for sequencing (http://www.tsingke.net) and the sequencing results verified by BLAST analysis (https://blast.ncbi.nlm.nih.gov/Blast.cgi). Using the primer of CVB5 amplified the 1078 bp fragment to further confirm our results. Finally, eleven pairs of primers were designed to perform PCR amplification of the whole genome segments of CVB5 isolates. The 5′-UTR primer of rhinovirus and CVB5 primer are shown in Table 1, eleven pairs of primers are shown in Table 2, and the five CVB5 sequences obtained were uploaded to the GenBank database [GenBank accession NOs. ON152867 (KM34), ON152868 (KM40), ON152869 (KM35), ON152870 (KM41), and ON152871 (KM48)].

**Table 1:** Primers for amplifying the 5′-UTR of rhinovirus and the VP1 genome sequence of coxsackievirus B5 strains [12]

Primer	Position	Sequence (5´ ~ 3´)	Product
5′-UTR-forward	140–157	CAAGCACTTCTGTTTCCC	394 bp
5′-UTR-reverse	515–533	CACGGACACCCAAAGTAGT	
VP1-forward	2344–2367	CCAAAGTGATTGCAAGATCTTGTG	
VP1-reverse	3399–3422	TGACTAGTAGGTCCCTGTTGTAAT	1078 bp

**Table 2:** Primers for amplifying the complete genome sequence of CVB5 strains

Primer	Position	Sequence (5´ ~ 3´)	Orientation
CVB5-F1	1–20	TTAAAACAGCCTGTGGGTTG	Forward
CVB5-R1	461–480	AGTTGGGATTAGCCGCATTC	Reverse
CVB5-F2	388–407	ATTYCGACATGGTGCGAAGA	Forward
CVB5-R2	948–967	TCCTCTGCTGACGGAGAGTT	Reverse
CVB5-F3	909–928	TCAARTCSATGCCTGCYCTC	Forward
CVB5-R3	1504–1523	RTTGATCCACTGRTGCGGGA	Reverse
CVB5-F4	1459–1478	GGTTGGWGTYGGCAATCTGA	Forward
CVB5-R4	2131–2150	AGCHCCTGGTGGTGARTACG	Reverse
CVB5-F5	2105–2124	AATGGCAACRGGYAAATTCC	Forward
CVB5-R5	2939–2958	AGAACACRCTAGGGTTGGTG	Reverse
CVB5-F6	2856–2875	TCATGTATGTGCCCCCGGGT	Forward
CVB5-R6	3581–3600	CGGCAAGCAARACATGTGTC	Reverse
CVB5-F7	3453–3472	CAACCGGGGTGTATTTYTGY	Forward
CVB5-R7	4254–4273	GCACTTTGCTCAATGGTGGC	Reverse
CVB5-F8	4134–4153	TACCGGARGTGARGGAGAAG	Forward
CVB5-R8	4893–4912	ATGGCCTTYCCRCACACGAG	Reverse
CVB5-F9	4801–4820	RRTCAACATGCCCATGTCAG	Forward
CVB5-R9	5746–5765	TCTCTTYGTGGGRGTGCCAC	Reverse
CVB5-F10	5642–5661	GCTGTYYTRGCWATAAACAC	Forward
CVB5-R10	6463–6482	TGTYTGYCTCATTGCHACWG	Reverse
CVB5-F11	6398–6417	CAGRTCWGCDGARAAGGTGG	Forward
CVB5-R11	7384–7403	CCGCACCGAATGCGGAGAAT	Reverse

The primers are located relative to the genome sequence of the 2013 Beijing strain (GenBank accession no. KY303900) reference strain

### Data analysis

On the basis of entire VP1 sequences of CVB5 available in GenBank, five sequences in this study and nine sequences isolate from fecal samples by our team’s previous work, the phylogeny of CVB5 was constructed [7]. In the phylogenetic tree, some sequences with the same country and temporal origin that were closely related were removed to obtain a simple and clear phylogenetic tree. The 55 reference sequences were screened to represent most of the major phylogenetic branches (A-E) [14]. MEGA 5.0 software was used to construct the phylogenetic tree using the N-J method. The nucleotide sequences of five CVB5 sputum isolates were analyzed using the homologous recombination software SimPlot. A comparative analysis of amino acid mutations was performed using BioEdit 7.09 software. The hydropathy changes in residues of VP1 protein between Faulkner and CVB5 isolates were analyzed using online prediction software Protscale (https://web.expasy.org/protscale/), PROVEAN approach was used to evaluate the possible structural and functional changes in the five sputum isolates VP1 protein compared to the Faulkner [15]. In PROVEAN, a threshold of − 2.5 was used (a score ≤ − 2.5 was considered deleterious, while a score > − 2.5 was considered neutral).

The possible VP1 protein structure models was predicted by Colabfold and predict protein mutation site in secondary structure by Lamdba Predict Protein (https://embed.predictprotein.org/). Then, the structural models of Faulkner and isolates were aligned using Pymol software to compare the differences of structure model.

## Results

### Case information

A total of five CVB5 complete genome sequences were obtained and the patient information of the five samples was collected and is shown in Table 3.

**Table 3:** Case description for pneumonia patients

Case description	Case 1 (KM34)	Case 2 (KM35)	Case 3 (KM40)	Case 4 (KM41)	Case 5 (KM48)
Sex	Female	Female	Male	Male	Male
Age	4 years	9 months	9 years	10 years	1 year
Clinical symptoms	fever, cough, wheeze	Fever, cough	Fever, cough	Cough	Cough
Peak body temperature	39.5 °C	39.5 °C	39.6 °C	36.4 °C	36.6 °C
Heart	Normal sounds upon auscultation	Normal sounds upon auscultation	Normal sounds upon auscultation	Normal sounds upon auscultation	Normal sounds upon auscultation
Lungs	Thick breath sounds of both lungs	Thick breath sounds of both lungs	Thick breath sounds of both lungs	Thick breath sounds of both lungs	Wheezing on auscultation
Throat	Slight congestion	Slight congestion	Slight congestion	Slight congestion	Slight congestion
Neck resistance	Normal	Normal	Normal	Normal	Normal
Abdomen	Soft	Soft	Soft	Soft	Soft
Liver and spleen	Impalpable	Impalpable	Impalpable	Impalpable	Impalpable
Hemoglobin level	135 g/L	111 g/L	126 g/L	132 g/L	135 g/L
WBC count	10.70 × 10^9^cells/L	6.97 × 10^9^cells/L	17.23 × 10^9^cells/L	4.01 × 10^9^cells/L	8.51 × 10^9^cells/L
RBC count	5.16 × 10^12^ cells/L	4.22 × 10^12^cells/L	/	4.71 × 10^12^ cells/L	4.85 × 10^12^cells/L
PLT count	295 × 10^9^cells/L	378 × 10^9^cells/L	574 × 10^9^cells/L	181 × 10^9^cells/L	235 × 10^9^cells/L
Causative agents of co-infections	HIN,ADV,SP,MP,PIV	PIV,HIN,SP	EBV,ADV,MP	MP,SP	MP

### Phylogenetic analysis

**Fig. 1:**
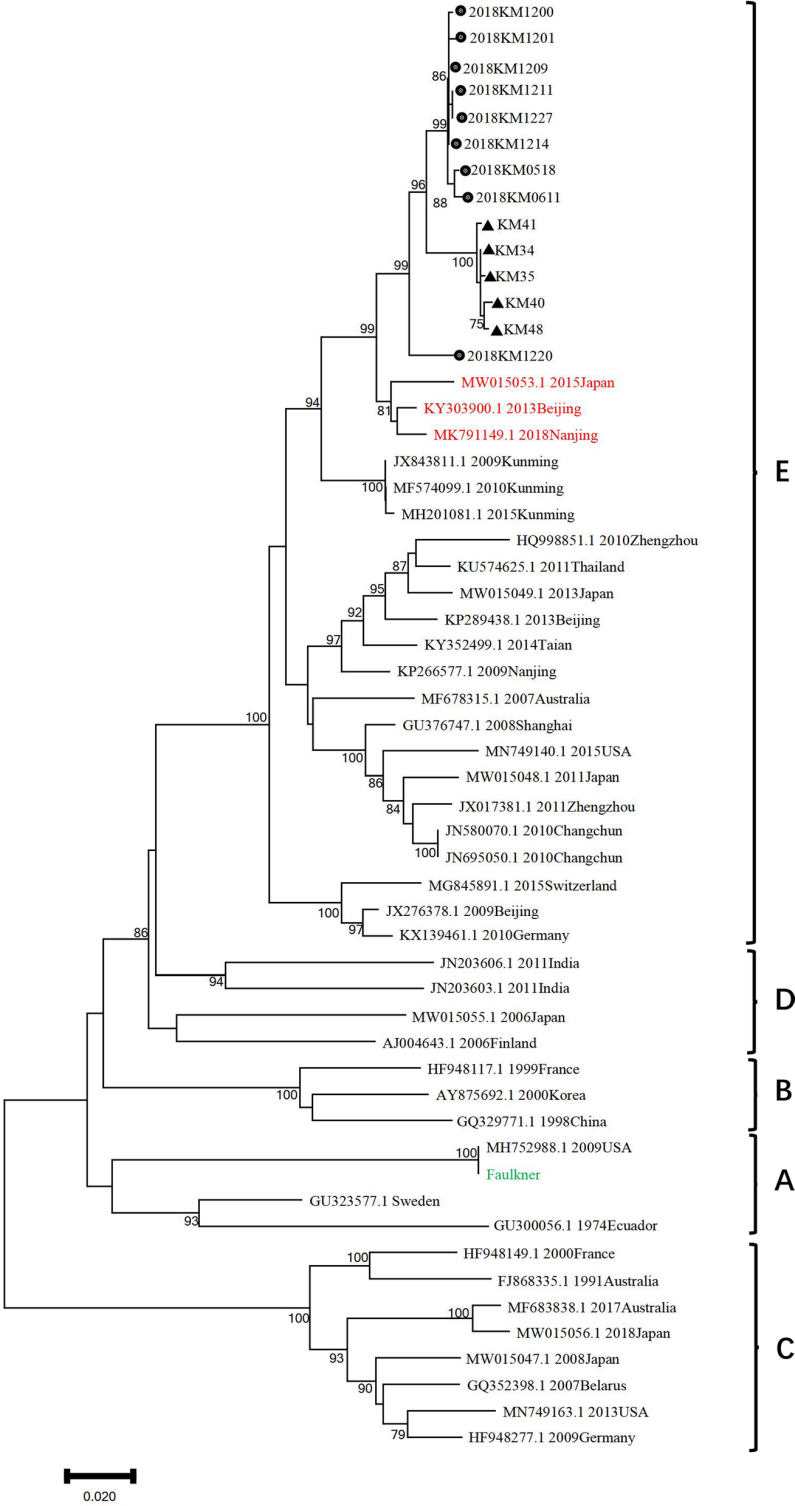
Phylogeny of CVB5 isolates based on the VP1 generated by the N-J method implemented in MEGA 5.0. The three isolates in red were highly similar to five sputum isolates and nine fecal isolates. The black circles indicate CVB5 isolates from fecal samples. The black triangles indicate CVB5 isolates from the sputum samples

Phylogenetic analysis of CVB5 VP1 sequences clustered all five sputum isolates into sublineage I in genogroup E (Fig. 1). They were in the same transmission chain as the fecal isolates collected in 2018. The five sputum isolates were highly similar to Nanjing 2018, Beijing 2013 and Japan 2015, average p-distance were 0.038, 0.041 and 0.048 respectively. The five sputum isolates in this article shared 8.7–12% sequence divergence with sublineage II strains in genogroup E. These results suggest that the five isolates in sputum and nine fecal isolates were form a independent branch in genogroup E [22].

### Complete genomic sequence and amino acid mutation analysis

**Fig. 2:**
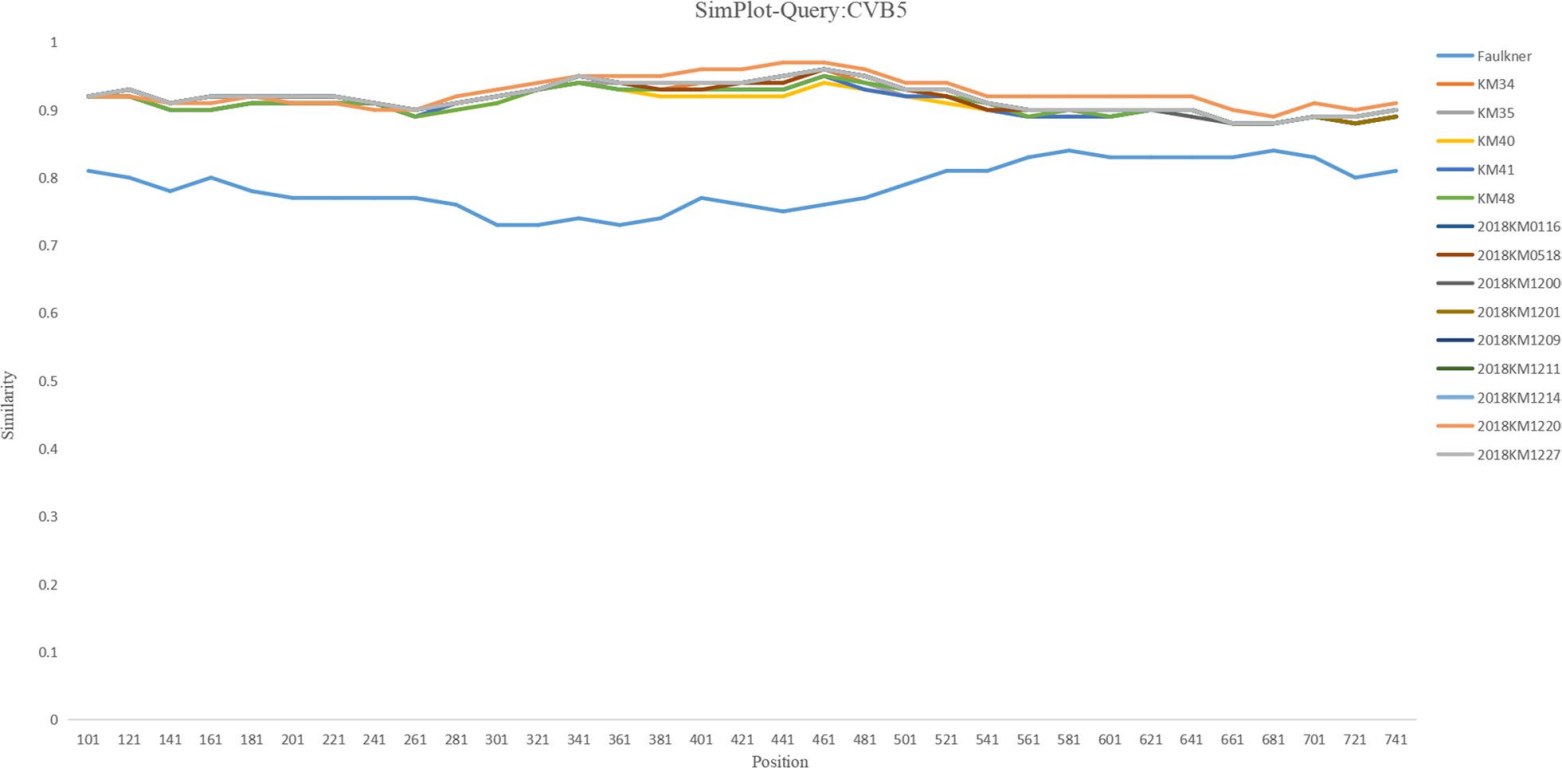
Similarity plot of the VP1 sequences of KM34, KM35, KM40, KM41 and KM48 compared to the Faulkner, and 2018 fecal isolates

**Fig. 3:**
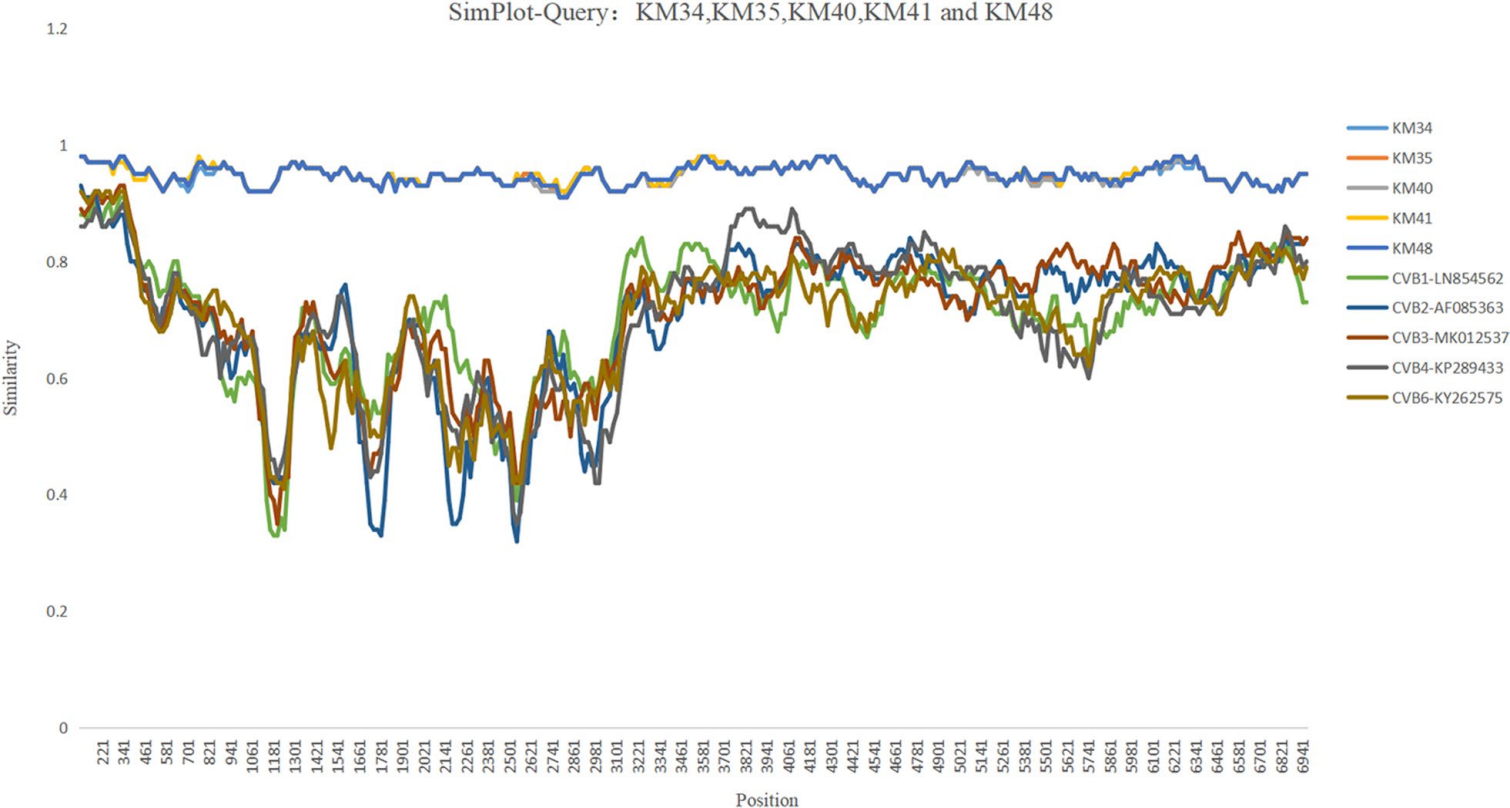
Similarity plot of the complete genome sequences of KM34, KM35, KM40, KM41 and KM48 comparing to other CVBs strains. Each point represents the similarity between the query sequence, with a 200-nt window moving in 20-nt steps. Positions containing gaps were excluded from the analysis

**Fig. 4:**
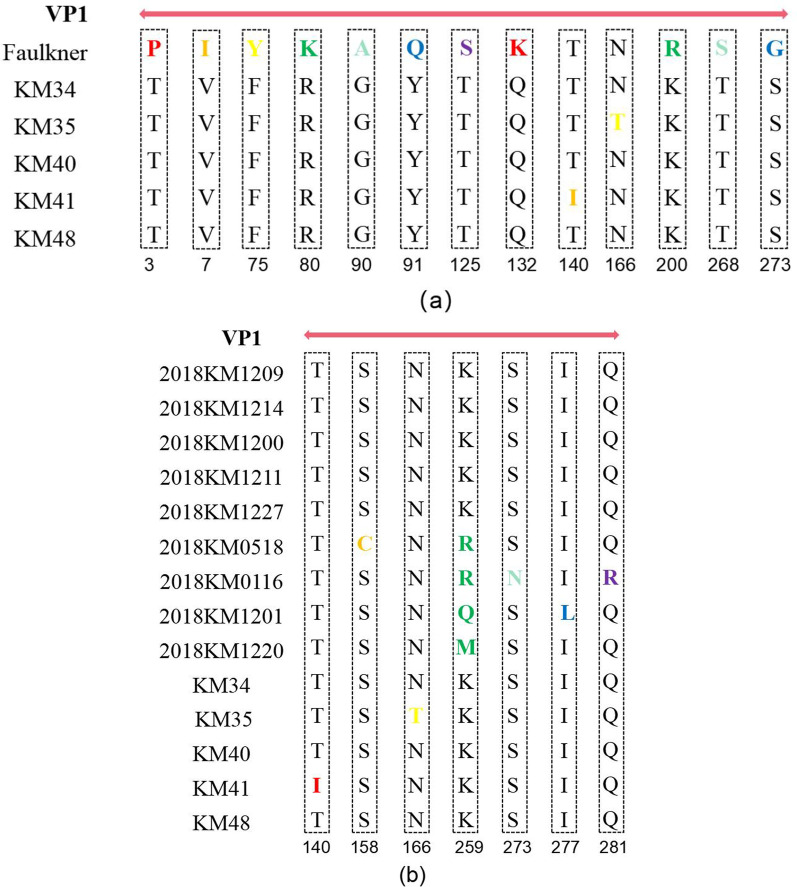
Amino acid mutations of the KM34, KM35, KM40, KM41, and KM48 isolates compared with the Faulkner (**a**) and 2018 fecal isolates (**b**)

The nucleotide and amino acid consistency of the five CVB5 isolates was 99.74–99.86% and 99.48–99.77%, respectively. Compared with Faulkner, the consistency of amino acids and nucleotides was 76.53–76.81% and 80.87–80.99%, respectively. In the VP1 region, the five sputum isolates were highly similar to the nine fecal isolates (Fig. 2). For the five sputum isolates, there were no obvious homologous recombination signals comparing with other coxsackie B viruses (Fig. 3). Eleven AA mutations at the 3th (C3: P → T), 7th (C7: I → V), 75th (C75: Y → F), 80th (C80: K → R), 90th (C90: A → G), 91th (C91: Q → Y), 125th (C125:S → T), 132th (C132: K → Q), 200th (C200: R → K), 268th (C268: S → T), 273th (C263: G → S) amino acids were observed in protein VP1 of the 5 isolates compared with Faulkner (Fig. 4a). An amino acid comparison with the fecal isolates showed that KM41 had a T-I (Thr-Ile) mutation at VP1 residue 140 and that KM35 had an N-T (Asn-Thr) mutation at residue 166 (Fig. 4b).

### Structural analysis and prediction

**Fig. 5:**
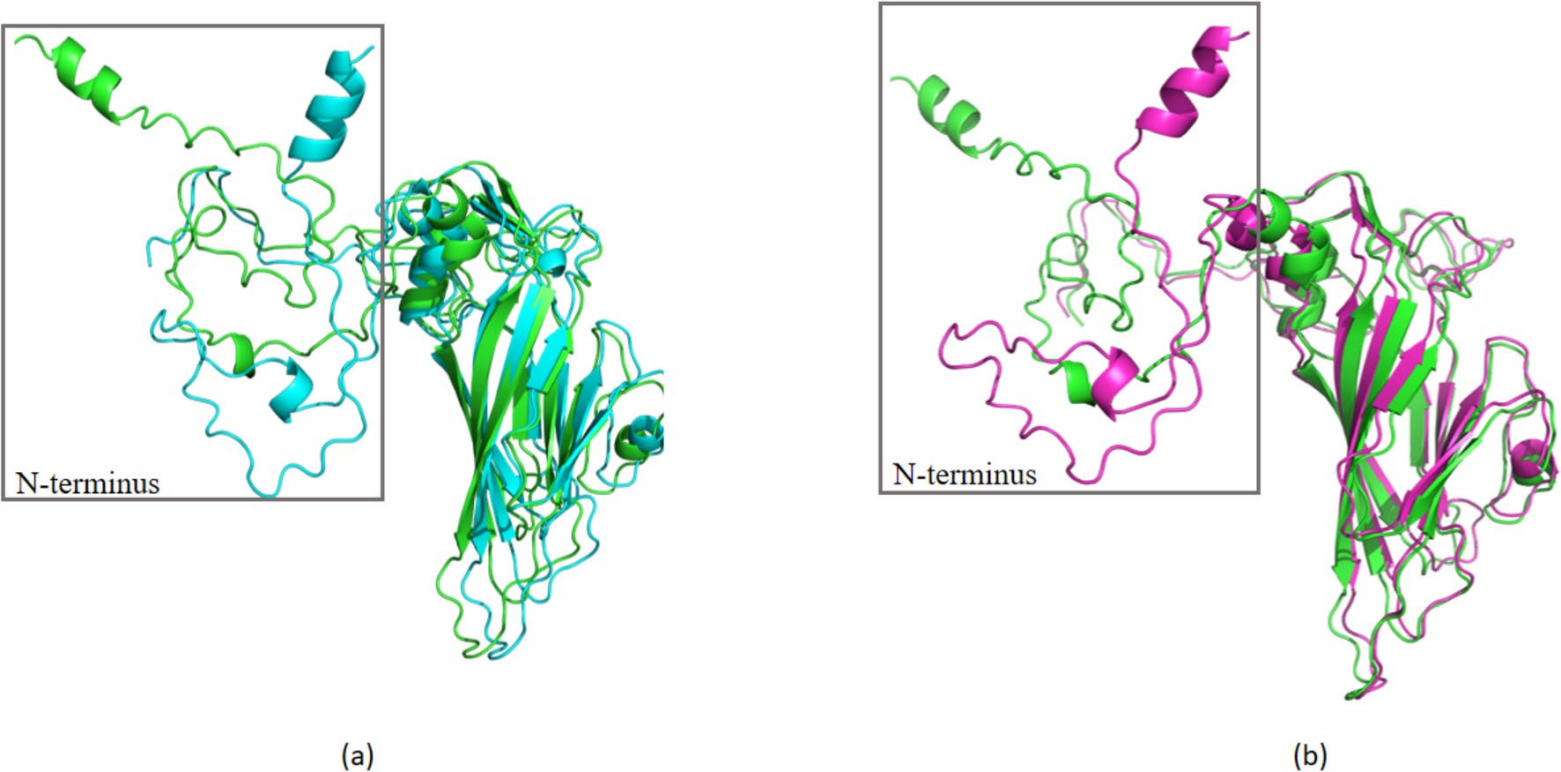
Aligned Faulkner, KM35 and KM41 with Pymol. Alignment diagram of Faulkner (green) and KM35 (blue). **a** Alignment diagram of Faulkner (green) and KM41 (red) (**b**)

Comparing to Faulkner, the amino acid alignment revealed 271 (96.0%) conserved and 12 (4.0%) variable positions in the VP1 protein. In addition, two substitutions in all five isolates were found in the BC loop region (K80R and A90G). The results of Protscale showed that the mutation of I7V, T140I (KM41) and N166T (KM35) significantly increased the hydrophobicity of the residues. Amino acid substitutions at the interaction interface may result in binding affinity changes. And the five sputum isolates VP1 amino acid substitutions comparing to Faulkner were investigated by the PROVEAN, which revealed three deleterious substitutions: Y75F, N166T (KM35) and T140I (KM41). These deleterious mutations may alter the structural stability of the protein (Table 4). Although these substitutions were considered deleterious, we can’t exclude the possibility of a milder infection or even a loss in viral fitness [15]. Aligned Faulkner, KM35 and KM41 with Pymol. Alignment diagram of Faulkner (green) and KM35 (blue) (Fig. 5a), the RMSD value is 0.276. Alignment diagram of Faulkner (green) and KM41 (red) (Fig. 5b), the RMSD value is 0.313. The smaller the RMSD, the higher the structural similarity of the protein.

**Table 4:** Changes and prediction of amino acid substitution effect of VP1 protein from five CVB5 isolates

VP1 amino acid residue		Prototype strain residue	Substitution	PROVEAN prediction
Position	Location			
3	N-terminus	P	P3T	Neutral
7	N-terminus(α-helix)	I	I7V	Neutral
75	β pleated sheet	Y	Y75F	Deleterious
80	BC-loop	K	K80R	Neutral
90	BC-loop	A	A90G	Neutral
91	β pleated sheet	Q	Q91Y	Neutral
125	DE-loop	S	S125T	Neutral
132	DE-loop	K	K132Q	Neutral
140*	β pleated sheet	T	T140I	Deleterious
166*	EF-loop	N	N166T	Deleterious
200	β pleated sheet	R	R200K	Neutral
268	C-terminus	S	S268T	Neutral
273	C-terminus	G	G273S	Neutral

*Position* Amino acid mutation site of VP1 protein compared to the prototype strain (Faulkner). *Location* The position of amino acid mutation site in VP1 protein secondary structure. BC Loop, DE-loop and EF-loop: The loop of antigen-antibody interaction. *Prototype strain residue* The amino acid residue in the prototype strain. *PROVEAN prediction* The PROVEAN algorithm result for the amino acid residue substitution

*It was not the common mutation site of the five isolates

## Discussion

Molecular epidemiological information on respiratory tract samples containing CVB5 is still limited. Under this background, we analyzed the molecular characteristics of CVB5 in patients with different symptoms, transmission and evolution of CVB5 in Kunming.

Phylogenetic and similarity analyses based on the VP1 region showed that the five sputum isolates were highly similar to the fecal isolates. However, there were nucleotide differences between the isolates and sublineage II of E genogroup (8.7–12%), the main strains endemic in Mainland China [18]. These information suggests that the five sputum isolates in 2021 and the nine fecal isolates in 2018 were in the same transmission chain and form an independent branch in genogroup E. The CVB5 isolated from patients with pneumonia sputum samples was not significantly different from the CVB5 we previously isolated in fecal samples from patients with HFMD in the VP1 region, but there were more mutations when comparing with Faulkner. Faulkner was isolated from patients in 1952 [20], nearly 70 years have passed between Faulkner and the CVB5 isolates in this study. The clinical symptoms of CVB5 may have changed due to the increasing differences in genomic sequences caused by the continuous evolution of the virus. In addition, the virus particles were successfully observed under an electron microscope in the harvested positive culture supernatants of KM35, and it will benefit the follow-up research of CVB5.

Amino acid mutations in VP1 were analyzed due to the lower homology of the isolates compared to Faulkner (80.87–80.99%). Four amino acid mutations were substituted at the N-terminus and C-terminus of the five isolates, and four mutations were in loop regions. The loops are located on the surface of the virion and are easily accessible to the host immune system [15]. Mutations at the N-terminal significantly changed the structure and spatial position of the N-terminal of VP1 protein. PROVEAN showed three deleterious mutations and nine neutral mutations. The deleterious mutations may lead to weakened virulence of the CVB5 isolates. Although neutral mutations can affect the binding of proteins, the main research direction is deleterious mutations, and the effect of neutral mutations needs to be further studied [2].

In China, patients with respiratory problems are often not tested for enterovirus to save on medical costs. The detection of enteroviruses in respiratory tract samples has also not received sufficient attention from clinicians. In the respiratory infection detection data from China over the past 11 years (2009–2019) that was released by the China CDC [13], enteroviruses were not monitored. The HFMD surveillance network established in China since 2009 is mainly based on clinical manifestations of diseases, and only several limited pathogens, such as EV-A71, CVA6, CVA10, and CVA16 are monitored [8]. These findings indicate a lack of enterovirus surveillance in patients with respiratory symptoms. CVB5 is the most common type among all coxsackie B viruses [21], but CVB5 has not even been incorporated into disease surveillance systems in China.

It was reported that the selective production of RANTES, IL-8 and MCP-1 by CVB5-infected epithelial cells of the small bronchioles, along with mechanisms of amplification mediated by IFN-γ [19]. This may be the various histologic and inflammatory features of CVB5-induced airway disease. The five patients in our study were all under the age of 10, and their common chief complaint before hospitalization was a prolonged cough. All five patients had almost the same symptoms as other pneumonia patients. Although CVB5 infection was detected in sputum samples from the five patients, they were co-infected with other pathogens. We can’t tell if CVB5 made their symptoms worse, constituted limiting factors in this investigation. In 1960, two children were reported to have died from pneumonia caused by CVB5 infection [3]. This suggests a certain mortality after CVB5 infection and should not be ignored in respiratory symptoms.

In conclusion, a total of 108 sputum samples from children hospitalized with lower respiratory tract infection were collected from the Children’s Hospital Affiliated with Kunming Medical University. We unexpectedly found five cases of CVB5 infection instead of rhinoviruses infection during our routine surveillance of rhinoviruses. This report suggests that enterovirus surveillance should be enhanced in patients with respiratory symptoms and serves as a reference to follow-up studies of molecular epidemiology, virulence, infection, and pathogenicity of CVB5.

## Data Availability

The datasets generated and analyzed during the current study are available from the corresponding author on reasonable request.

## Abbreviations

A90G: Replacement of alanine by glycine at position 90 of the VP1 protein
ADV: Adenovirus
BLAST: Basic local alignment search tool
CAR: Coxsackievirus and adenovirus receptor
CDC: Centers for disease control
CVA6: Coxsackievirus A6
CVA10: Coxsackievirus A10
CVA16: Coxsackievirus A16
CVB5: Coxsackievirus B5
DAF: Decay accelerating factor
EBV: Epstein-Barr virus
EV-A71: Enterovirus A71
G273S: Replacement of glycine by serine at position 273 of the VP1 protein
HFMD: Hand, foot and mouth disease
HIN: Haemophilus influenzae
I7V: Replacement of isoleucine by valine at position 7 of the VP1 protein
K80R: Replacement of lysine by arginine at position 80 of the VP1 protein
K132Q: Replacement of lysine by glutarnine at position 132 of the VP1 protein
MP: Mycoplasma pneumoniae
N166T: Replacement of asparagine by threonine at position 166 of the VP1 protein
N-J: Neighbor-joining
P3T: Replacement of proline by threonine at position 3 of the VP1 protein
PIV: Parainfluenza virus
PLT: Platelet
PROVEAN: Protein variation effect analyzer
Q91Y: Replacement of glutarnine by tyrosine at position 91 of the VP1 protein
R200K: Replacement of arginine by lysine at position 200 of the VP1 protein
RBC: Red blood cell
RT-PCR: Reverse transcription polymerase chain reaction
RMSD: Root mean square deviation
S125T: Replacement of serine by threonine at position 125 of the VP1 protein
S268T: Replacement of serine by threonine at position 268 of the VP1 protein
SP: Streptococcus pneumoniae
T140I: Replacement of threonine by isoleucine at position 140 of the VP1 protein
UTR: Untranslated regions
VP1-4: Viral protein 1-4
WBC: White blood cell
Y75F: Replacement of tyrosine by phenylalanine at position 75 of the VP1 protein
